# Result of type a dissecting aortic aneurysms surgical treatment during the 3 years (2010 - 2012)

**DOI:** 10.1186/1749-8090-8-S1-O25

**Published:** 2013-09-11

**Authors:** VI Kravchenko, IM Kravchenko, LL Sytar, OA Tretyak, OB Larionova, OV Rybakova, YM Tarasenko, GV Knyshov

**Affiliations:** 1 Department of Surgical Treatment of Aortic Pathology, National M. Amosov ICVS NAMS of Ukraine, Kyiv, Ukraine; 2 Department of Anaestesiology, National M. Amosov ICVS NAMS of Ukraine, Kyiv, Ukraine; 3 ICU of Department Surgical Treatment of Aortic Pathology, National M. Amosov ICVS NAMS of Ukraine, Kyiv, Ukraine; 4 Scientific Secretariat, National M. Amosov ICVS NAMS of Ukraine, Kyiv, Ukraine; 5 Director of National M. Amosov ICVS NAMS of Ukraine, Kyiv, Ukraine

## Background

Present our experience of surgical treatment of type A TAAD during 2010 – 2012 - the aim of this study.

## Methods

148 consecutive patients with type A TAAD were operated on during 2010 - 2012 (122 (82,4%) males). Their age ranged 20 – 77 years, mean 49,3±9,6. Acute (subacute) dissection was in 128 (86,5%), chronic – in 20 (13,5%). The causes of aneurysms forming were: arterial hypertension, atherosclerosis – in 91 (61,5%); BAV – 18 (12,2%); MS – 16 (9,8%); cystomedianecrosis – 16 (10,8%); blunt aortic injury – in 2 (1,4%) cases, unknown – 5 (3,4%); 96 (64,8%) patients had type I, others 52 (35,2%) – type II according De Bakey classification. The preoperative status included: acute aortic valve insufficiency - 64 (43,2%); haemopericardium (heart tamponade) - 26 (17,6%); acute renal insufficiency - 12 (8,1%); left ventricle failure with pulmonary edema - 5 (3,4%); multiorgan failure - 4 (2,7%) patients.

All operations were performed with bypass, mild hypothermia (26-30ºC), 42 (28,4%) patients with arch injury - deep hypothermia (18-20ºC) and retrograde cerebral perfusion.

We used: supracoronary grafting with valve resuspension- in 98 (66,2%) pts, Bentall-de Bono operation in 48 (32,4%); David operation in 2 (1,4%).

## Results

Mean blood loss after operation was 519±82,7 ml. Hemorrhage became the reoperation reason in 5 (3,4%) pts. Temporary neurological complications were observed in 7 (4,7%) pts. There were no difference in deep and mild hypothermia group. Permanent neurological complications were absent. The postoperative 30 days mortality composed 4,7% (7 patients). All lethal events occurred in the acute dissection group. The reasons of lethal events were: acute renal failure – in 4 (2,7 %) patients, hemorragie – in 1(0,7%) patients, multiorgan failure – in 2 (1,3%).

## Conclusion

Obtained surgical experience, improvement of heart and brain protection in surgical treatment of dissecting aneurysms type A permitted to achieve hospital mortality 4,7%.

